# Electroretinogram Changes following Silicone Oil Removal

**Published:** 2011-04

**Authors:** Mohsen Azarmina, Masoud Soheilian, Hossein Azarmina, Bagher Hosseini

**Affiliations:** Ophthalmic Research Center, Labbafinejad Medical Center, Shahid Beheshti University of Medical Sciences Tehran, Iran

**Keywords:** Vitrectomy, Silicone Oil, Electroretinogram

## Abstract

**Purpose:**

To evaluate electroretinogram (ERG) changes after silicone oil removal.

**Methods:**

Scotopic and photopic ERGs, and best-corrected visual acuity (BCVA) were checked before and shortly after silicone oil removal in eyes that had previously undergone vitrectomy and silicone oil injection for complex retinal detachment. Pre- and postoperative ERG a- and b-wave amplitudes were compared.

**Results:**

Twenty-eight eyes of 28 patients including 20 male and 8 female subjects with mean age of 39.3 ± 0.06 (range, 12 to 85) years were studied. Mean interval from primary vitreoretinal surgery to silicone oil removal was 21.04 ± 0.52 (range, 7 to 39) months. Mean duration from silicone oil removal to second ERG was 13.04 ± 1.75 (range, 10 to 16) days. Before silicone oil removal, mean a-wave amplitudes in maximal combined response, rod response and cone response ERGs were 27.4 ± 19.9, 7.2 ± 4.5 and 5.5 ± 3.4 μv, respectively. These values increased to 48.8 ± 31.9, 15.1 ± 14.4 and 17.4 ± 22.2 μv, respectively after silicone oil removal (P < 0.001). Mean b-wave amplitudes in the same order, were 69.41 ± 51, 41.2 ± 30.4 and 25.1 ± 33.9 μv before silicone oil removal, increasing to 165.6 ± 102.5, 81.7 ± 53.7 and 44.7 ± 34.1 μv respectively, after silicone oil removal (P < 0.001). Mean BCVA significantly improved from 1.10 ± 0.34 at baseline to 1.02 ± 0.33 logMAR after silicone oil removal (P < 0.001).

**Conclusion:**

The amplitudes of ERG a- and b-waves under scotopic and photopic conditions increased significantly shortly after silicone oil removal. An increase in BCVA was also observed. These changes may be explained by the insulating effect of silicone oil on the retina.

## INTRODUCTION

Silicone oil was introduced by Cibis and associates for intraocuar tamponade in the surgical management of complicated retinal detachments.^1^,^2^ It has been used successfully for long term tamponade and maintenance of reattachment in complex retinal detachments (RDs), severe proliferative vitreoretinopathy (PVR), and RDs following severe trauma. The use of silicone oil in conjunction with vitrectomy is considered as the last resort for treatment of severe proliferative diabetic retinopathy (PDR).^3^,^4^ With the improved understanding of vitreoretinal abnormalities and advanced surgical techniques, silicone oil is being used extensively for many retinal disorders.^4^–^8^ The use of silicone oil for temporary internal tamponade in our patients provided us a chance to study the insulating effect of silicone oil on the retina by electroretinography (ERG) and to compare its changes after removal of the oil.

## METHODS

Twenty-eight eyes of 28 patients, including 20 male and 8 female subjects with mean age of 39.3±0.06 (range, 12 to 85) years with complex RD who had undergone vitrectomy and temporary tamponade with purified silicone (5000 centistokes) were studied. Minimum follow-up after surgery was 3 months. Patients with vascular eye disease such as diabetic retinopathy or retinal vascular accidents, pre- or postoperative glaucoma, media opacity (corneal or dense cataract), and complicated silicone removal were excluded from the study. Uneventful silicone oil removal was considered essential for comparison of pre- and postoperative electrophysiological results. The retina was attached before silicone oil removal in all cases.

Photopic (cone response), scotopic (rod response) and maximal combined response ERGs were obtained according to the methods described by the International Society for Clinical Electrophysiology of Vision (ISCEV) using the Mono Elec 2 system (Metrovision Inc., France) in all cases before and shortly after silicone oil removal. The amplitude of a- and b-waves before and after silicone oil removal in conditions of rod response, maximal combined response and cone response were detected and compared using Wilcoxon test with significance set at P<0.05.

## RESULTS

Table 1 summarizes demographic and clinical data of the patients. Underlying conditions leading to vitrectomy and internal tamponade with silicone oil were traumatic RD in 9 (32.1%), pseudophakic RD in 8 (28.6%), myopic RD in 7 (25.9%), and aphakic RD, macular hole RD, giant retinal tear and stickler syndrome, each in one (3.57%) case. The retina remained attached after silicone oil removal in all 28 patients. Mean duration of silicone oil retention was 21.04±0.06 (range, 7 to 39) months. Mean duration from silicone oil removal to the second ERG recording was 13.04±1.75 (range, 10 to 16) days.

**Figure 1:**
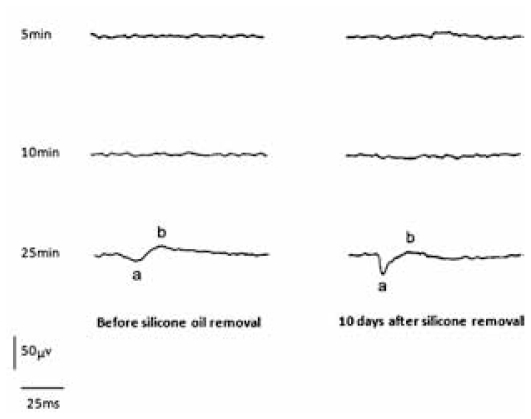
A representative case (No:6) demonstrates minimal response ERG; despite increasing dark adaptation from 5 minutes to 10 and 25 minutes and use of a high intensity stimulus, no significant electroretinographic amplitude could be recorded before silicone oil removal. ERG in the same patient 10 days after silicone oil removal demonstrates a significant increase in both a- and b-wave amplitudes with the use of a high-intensity stimulus and progressive dark adaptation.

The amplitudes of ERG a- and b-waves were greatly reduced or even unrecordable before silicone oil removal. The amplitudes of both waves increased significantly after silicone oil removal (Fig. 1), although the anatomic condition and retinal attachment status remained unchanged.

Mean BCVA before silicone oil removal was 1.10±0.34 (range, 0.04 to 1.70) logMAR which significantly improved 1.02±0.33 (range, 0.04 to 1.70) logMAR afterwards, indicating a mean difference of −0.08±0.09 logMAR (95% confidence interval, −0.11 to −0.03; P<0.001).

Tables 2 and 3 compare a- and b-wave amplitudes before and after silicone oil removal under conditions of maximal combined, rod and cone response respectively; all changes were statistically significant (P<0.001) in all conditions.

## DISCUSSION

Histologic and electrophysiologic findings following vitreoretinal surgery utilizing silicone oil tamponade seem contradictory and different conclusions have been made on the toxicity of liquid silicone.^9^–^10^ In enucleated eyes after silicone surgery, vacuole formation has been observed in the retina and optic nerve, and even beyond the lamina oribrosa.^11^ In animal experiments, vacuole formation and destruction of ganglion cells have been described following silicone injection.^9^–^12^ Although cataract formation, keratopathy and secondary glaucoma are well known complications of silicone oil filling,^8^,^13^ the question of retinal toxicity still remains unanswered.

Most investigators have reported no histological evidence of retinal damage due to silicone oil. All agree that in silicone oil filled eyes, ERG and EOG (electrooculogram) waves are diminished or even unrecordable, but they have different interpretations for these finding. Some authors^9^,^12^,^14^ consider them as a sign of retinal toxicity, while others^3^,^15^,^16^ have assumed an insulator effect by silicone oil. The insulating effect of silicone oil on the retina was confirmed in our study such that the amplitude of ERG a- and b-waves under both photopic and scotopic conditions was either low or unrecordable before silicone oil removal but increased significantly in all patients; a finding which is in line with other published reports.^17^,^18^

Regarding the minimum duration of four weeks for photoreceptor recovery after retinal reattachment,^19^,^20^ silicone oil was removed 7 to 39 months after vitreoretinal surgery in our series. We assumed that by this time, the photoreceptors should have made ultimate recovery and ERG amplitudes could be considered as a reliable baseline for comparison after silicone oil removal.^19^,^20^

The baseline level of the corneoretinal potential was low in all eyes before silicone oil removal but increased afterwards. The reduced baseline level of the corneoretinal potential in silicone oil filled eyes is due to an insulating effect and equivalent to reduction of ERG amplitudes. A similar insulating effect has been reported in gas-filled eyes as long as the gas is retained within the eye.^17^

We may conclude that silicone oil removal increases ERG wave amplitudes and improves visual acuity after an adequate period of retention necessary for establishment of retinal reattachment and photoreceptor recovery. These changes in ERG a- and b-wave amplitudes may be attributed to the insulating effect of silicone oil on the retina rather than actual toxicity. Silicone oil should therefore be removed from all eyes after accomplishment of its effect.

## Figures and Tables

**Table 1:** Demographic and clinical data of the patients before and after silicone oil removal

No	Age (years)	Sex	Eye	Underlying disease	Duration of retained silicone oil (months)	Duration from oil removal to ERG (days)	BCVA		a-wave amplitude (μv)						b-wave amplitude (μv)					

									Maximal combined response		Rod response		Cone response		Maximal combined response		Rod response		Cone response	
	
							Pre	Post	Pre	Post	Pre	Post	Pre	Post	Pre	Post	Pre	Post	Pre	Post
1	69	F	OD	Myopic RRD	23	14	20/200	20/160	28.1	99.2	3.1	14.1	7	6	93.7	244	59.3	144	24.3	84.7

2	54	F	OS	Myopic RRD	16	16	CF3m	CF3m	17.2	26.6	10.8	7.7	6.4	8.7	24.9	63.2	11.7	17.2	13.5	14.6

3	28	M	OS	Traumatic RRD	7	13	CF2.5m	CF3m	47.7	78	10.2	15	10.2	12.3	84.3	205	50.7	98.3	28	54.1

4	26	M	OS	Traumatic RRD	8	14	20/160	20/160	43	50	10.2	8.7	6	18.9	93.7	312	55.4	90	26.4	53.7

5	21	F	OS	Pseudophakic RRD	25	13	20/200	20/120	1.6	3.8	6.3	7	1	5.4	10.9	10.8	7.7	7	18.5	14.9

6	12	M	OS	Myopic RRD	19	12	CF4m	CF4m	14.1	37.5	11.7	3.1	5.2	7.4	31.2	37.5	14	14	5.6	7.5

7	31	F	OD	Myopic RRD	30	13	20/160	20/120	7	16.4	0.8	1.2	4.3	4.5	17.9	38.2	8.5	11.6	7.6	19

8	30	M	OD	Traumatic RRD	28	14	20/50	20/50	46.1	67.2	7.8	10.2	16.2	14.2	198	266	108	104	44.9	56.2

9	37	M	OS	Aphakic RRD	32	15	CF3m	CF4m	9.4	66.2	1.5	68.8	2.2	8.1	21.8	66.2	5.5	92.9	4.3	9.8

10	52	M	OS	Traumatic RRD	36	15	CF2m	CF2.5m	23.3	6.3	6.9	5.6	2.6	1.2	84.4	64.6	42.2	39.7	9	5.7

11	33	F	OD	Retinal angiomatosis	27	14	CF1.5m	CF2m	37.5	39.1	5.5	5.5	7.9	6.3	94.4	148	53	93.7	24.1	21.2

12	60	M	OD	Pseudophakic RRD	39	12	CF3m	CF3m	20.3	23.4	3.1	6.3	6.3	120	35.8	294	16.3	20.5	15.2	152

13	54	M	OS	Pseudophakic RRD	21	13	20/200	20/120	22.7	68	0	1.5	2.6	23.2	31.9	121	28	105	17.4	13.5

14	16	M	OS	Myopic RRD	15	11	20/160	20/160	15.6	58.6	12.5	7.8	6.3	10.5	37.4	121	18.7	46	13.9	41.5

15	32	M	OS	Myopic RRD	18	10	CF1m	CF1.5m	14	39.1	11.7	43.8	2.1	9.1	15.6	213	68.7	138	22.9	49.2

16	47	M	OS	Pseudophakic RRD	31	13	20/160	20/160	3.9	16.2	6.3	16	8.7	40.8	34.3	112	21	69.4	24.9	61.2

17	85	M	OS	Traumatic RRD	17	16	CF1m	CF2m	76	91.4	0.8	13.3	5.2	19.1	112	249	66.3	130	30	90.9

18	38	F	OS	Stickler syndrome	20	13	20/200	20/160	45.8	43	0	28.1	7.2	14.7	166	173	105	118	27	32.8

19	19	M	OS	Traumatic RRD	8	13	20/200	20/120	42.2	56.3	12	12.5	4.3	28.9	106	202	74	113	190	58.4

20	50	M	OD	Pseudophakic RRD	28	14	20/200	20/200	32.8	58.6	14.1	20.3	5.6	8.3	60.8	176	54.6	108	7.8	32.2

21	36	M	OS	Macular hole RD	25	11	CF5m	20/160	10.4	14.8	0.7	2.3	1.7	3.9	13.6	98.4	6.3	23	1.4	17

22	38	F	OS	Traumatic RRD	15	15	20/160	20/160	75	149	14.1	21.1	8.3	23.2	112	318	59.3	173	27.7	81.7

23	39	M	OS	Pseudophakic RRD	12	12	20/160	20/120	45.3	73.4	7	35.2	9.4	34.1	162	326	79.6	208	32.4	99.6

24	43	F	OD	Myopic RRD	29	12	CF5m	CF5m	34.4	43.8	7.7	15.6	0.6	8.1	28.1	49.9	5.5	24.2	17.2	23.3

25	39	M	OD	Pseudophakic RRD	17	14	CF2m	CF2.5m	7.7	24.9	8.1	11	2.2	7.9	12.5	35.9	3	10.9	2.1	17

26	55	M	OS	RRD	19	10	20/80	20/80	26.6	39.1	6	8.6	2.6	11.8	65.5	98.3	26.5	72.6	18.8	26.1

27	27	M	OS	Traumatic RRD	11	13	20/160	20/160	4.3	59.1	10.3	12.5	6	18.8	93.2	315	55.4	98	26.2	54.5

28	30	M	OD	Traumatic RRD	13	10	20/60	20/60	15	17.9	12.5	18.7	6.8	12.7	91	278	49	117	23	58

F, female; M, male; OD, right eye; OS, left eye; RRD, rhegmatogenous retinal detachment; BCVA, best-corrected visual acuity; CF, counting fingers; Pre, before silicone oil removal; Post, after silicone oil removal.

**Table 2:** a-wave amplitude (microvolts) before and after silicone oil removal

Response	Mean ± Standard Deviation (range)			95% CI of difference	P value
	Pre	Post	Difference		
Maximal combined	27.4 ± 19.9 27 (1.6 to 76)	48.8 ± 31.9 43.4 (3.8 to 149)	21.4 ± 22.4 14.8 (−17 to 74)	12.7 to 30.1	<0.001
Rod	7.2 ± 4.5 7.4 (0 to 14.1)	15.1 ± 14.4 11.8 (1.2 to 68.8)	7.8 ± 15.1 2.8 (−8.6 to 67.3)	−2 to 13.7	0.001
Cone	5.5 ± 3.4 5.8 (0.6 to 16.2)	17.4 ± 22.2 11.2 (1.2 to 120)	11.9 ± 21.8 5.9 (−2 to 113.7)	3.5 to 20.3	<0.001

Pre, before silicone oil removal; Post, after silicone oil removal; CI, confidence interval

**Table 3:** b-wave amplitude (microvolts) before and after silicone oil removal

Response	Mean ± Standard deviation (range)			95% CI of difference	P value
	Pre	Post	Difference		
Maximal combined	69.0 ± 51.0 63.2 (10.9 to 198)	165.6 ± 102.5 160.5 (10.8 to 326)	96.5 ± 78.5 84.2 (−19.8 to 258.2)	66.1 to 127	<0.001
Rod	41.2 ± 30.4 45.6 (3.0 to 10.8.0)	81.7 ± 53.7 93.3 (7.0 to 208.0)	40.5 ± 36.1 39.9 (−4 to 128.4)	26.5 to 54.4	<0.001
Cone	25.1 ± 33.9 20.9 (1.4 to 190)	44.7 ± 34.1 37.2 (5.7 to 152)	19.5 ± 42.1 15.2 (−131.6 to 136.8)	3.2 to 35.8	<0.001

Pre, before silicone oil removal; Post, after silicone oil removal; CI, confidence interval
